# Chimeric cerebral organoids reveal the essentials of neuronal and astrocytic *APOE4* for Alzheimer’s tau pathology

**DOI:** 10.1038/s41392-022-01006-x

**Published:** 2022-06-13

**Authors:** Shichao Huang, Zhen Zhang, Junwei Cao, Yongchun Yu, Gang Pei

**Affiliations:** 1 State Key Laboratory of Cell Biology, Shanghai Institute of Biochemistry and Cell Biology, Center for Excellence in Molecular Cell Science, Chinese Academy of Sciences, 200031 Shanghai, China; 2 Institutes of Brain Science, State Key Laboratory of Medical Neurobiology and Collaborative Innovation Center for Brain Science, Fudan University, 200032 Shanghai, China; 3 Shanghai Key Laboratory of Signaling and Disease Research, Laboratory of Receptor-based Biomedicine, The Collaborative Innovation Center for Brain Science, School of Life Sciences and Technology, Tongji University, Shanghai, China; 4 Institute for Stem Cell and Regeneration, Chinese Academy of Sciences, Beijing, China

**Keywords:** Neurological disorders, Pluripotent stem cells

## Abstract

The apolipoprotein E4 (*APOE4*) genotype is one of the strongest genetic risk factors for Alzheimer’s disease (AD), and is generally believed to cause widespread pathological alterations in various types of brain cells. Here, we developed a novel engineering method of creating the chimeric human cerebral organoids (chCOs) to assess the differential roles of *APOE4* in neurons and astrocytes. First, the astrogenic factors NFIB and SOX9 were introduced into induced pluripotent stem cells (iPSCs) to accelerate the induction of astrocytes. Then the above induced iPSCs were mixed and cocultured with noninfected iPSCs under the standard culturing condition of cerebral organoids. As anticipated, the functional astrocytes were detected as early as 45 days, and it helped more neurons matured in chCOs in comparation of the control human cerebral organoids (hCOs). More interestingly, this method enabled us to generate chCOs containing neurons and astrocytes with different genotypes, namely *APOE3* or *APOE4*. Then, it was found in chCOs that astrocytic *APOE4* already significantly promoted lipid droplet formation and cholesterol accumulation in neurons while both astrocytic and neuronal *APOE4* contributed to the maximum effect. Most notably, we observed that the co-occurrence of astrocytic and neuronal *APOE4* were required to elevate neuronal phosphorylated tau levels in chCOs while Aβ levels were increased in chCOs with neuronal *APOE4*. Altogether, our results not only revealed the essence of both neuronal and astrocytic *APOE4* for tau pathology, but also suggested chCOs as a valuable pathological model for AD research and drug discovery.

## Introduction

Alzheimer’s disease (AD) is the most common kind of dementia that is characterized by deteriorating memory and other cognitive functions.^1^ In AD, the aggregation of amyloid-β (Aβ) and phosphorated microtube-associated protein tau leads to the formation of senile plaques and neurofibrillary tangles (NFT), which are considered as two major neuropathological hallmarks of AD.^2^ These pathogenic aggregates of Aβ and tau induce the destruction of synapses, thus cause memory and cognitive impairment during the early phase of AD pathogenesis.^3^ The majority (>95%) of AD cases are late-onset sporadic AD (LO-SAD) and <5% are early-onset familial AD (EO-FAD).^4^ Accumulating evidences indicate that EO-FAD is associated with various mutations in genes linked to Aβ biogenesis, such as the amyloid precursor protein (*APP*) and the presenilins (*PS1* and *PS2*), suggesting abnormal Aβ metabolism in EO-FAD.^5^ Compared with EO-FAD, the etiology of LO-SAD is likely to be more complicated and much less clear. A number of genetic factors are reported to be associated with LO-SAD, including but not limited to apolipoprotein E *(APOE*), clusterin (*CLU*), phosphatidylinositol binding clathrin assembly protein (*PICALM*), triggering receptor expressed on myeloid cells 2 (*TREM2*), complement component 3b/4b receptor 1 (CR1), ATP-binding cassette transporter member 7 (*ABCA7*), bridging integrator 1 (*BIN1*), and sialic acid binding Ig-like lectin 3 (*CD33*). Among them, *APOE* is the highest ranked susceptibility gene for LO-SAD.^6^

*APOE* gene is strongly associated with the onset of Alzheimer’s disease. There are three polymorphic alleles of *APOE*, namely *APOE2, APOE3*, and *APOE4*. Compared with the *APOE3* homozygotes, people carrying two copies of *APOE4* have a roughly 14-fold increase in the risk of developing AD, while *APOE2* allele is protective.^7^ ApoE is an apolipoprotein, which play critical roles in cholesterol transport and lipid homeostasis.^8,9^ In the brain, ApoE is mainly synthesized and secreted by astrocytes, but is also expressed by other brain cell types including neurons.^10,11^ Studies demonstrate lipid metabolic defects and mitochondrial dysfunction in *APOE4* astrocytes, which has a positive correlation of AD pathogenesis.^12–14^ Additionally, astrocytic-specific removal of *APOE4* reduced tau-mediated neurodegeneration and phosphorylated tau (p-tau) pathology.^15^ Similarly, *APOE4* neurons also display multiple AD-linked molecular and cellular alterations, including increased levels of p-tau, diminished synaptic plasticity, and cholesterol dysregulation.^13,16^ Although the role of *APOE4* on different brain cell types has been studied separately, the overall effects of different *APOE4*-carrying cell types on AD pathogenesis remain largely unexplored. A recent study on mouse models with different humanized *APOE* alleles has revealed that *APOE4* astrocytes cause disease-associated changes on various brain cells including neurons, oligodendrocytes, and microglia.^15^ Be aware of the anatomical and physiological differences between humans and animals, it is necessary to evaluate the effects of different *APOE4* cells in human cell-based disease models in parallel with animal studies.

Human cerebral organoids (hCOs) recapitulate architectural and functional features of human brain and provide good in vitro models for studying neurological disorders. Astrocytes play important roles in modulating neurogenesis, synapse formation and metabolic homeostasis.^17–19^ Impairment in astrocytes implicates pathogenesis of various neurodegenerative diseases including Alzheimer’s disease, Huntington disease, and ischemic stroke. However, the astrocytic differentiation in hCOs is much slower compared with neuronal differentiation^20,21^ The lack of astrocytes not only delays hCO maturation, but also limits their application in disease modeling.^22^

To assess the effect of neuronal and astrocytic *APOE4* on different AD pathologies, we generated chimeric hCOs (chCOs) with induced astrocytes by ectopically expressing the transcription factors NFIB and SOX9 in part of hCOs. We further generated four kinds of chCOs with different *APOE* genotypes in neurons and astrocytes, respectively. We showed that *APOE4* astrocytes elevated neuronal lipid droplet formation and cholesterol accumulation. Notably, we also found that the co-occurrence of *APOE4* astrocytes and neurons were required for the elevation of neuronal p-tau levels in chCOs, indicating the essentials of both neuronal and astrocytic *APOE4* for the tau pathology.

## Results

### Accelerated induction of astrocytes in iPSC‑derived chCOs

**Fig. 1:**
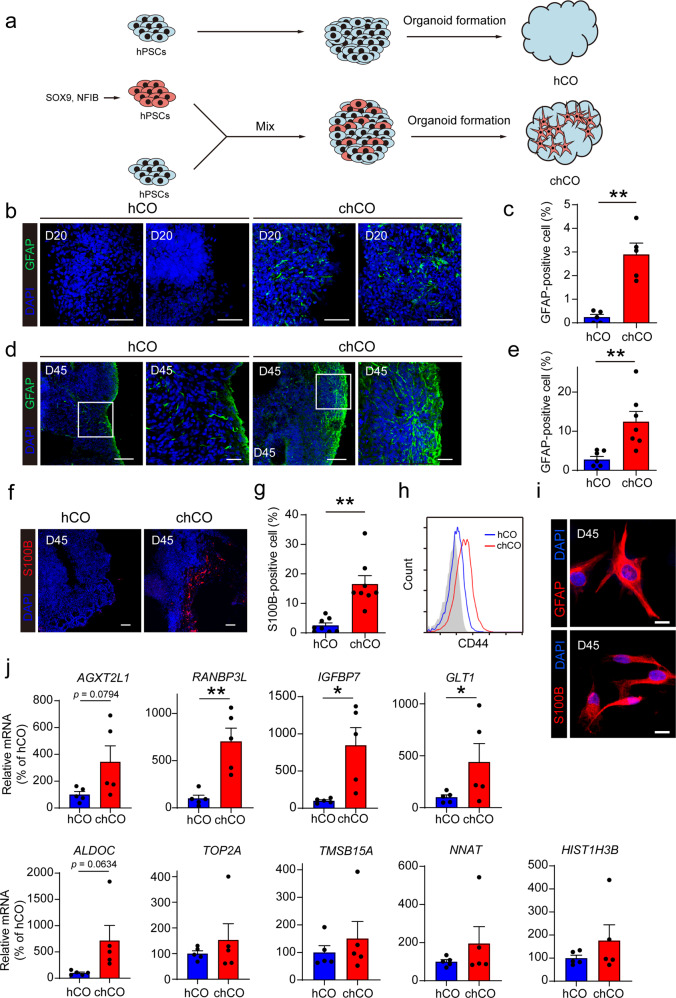
Induction of astrocytes in human cerebral organoids by Sox9 and NFIB expression. **a** Experiment procedure for generation of chCOs and control hCOs. **b**, **c** Immunostaining of chCOs and control hCOs on D20 for GFAP. Shown in **c** is the quantification of the percentage of the GFAP-positive area over the whole organoid area. Data represent the mean ± s.e.m. (*n* = 5 organoids, from three different batches; **p < 0.01). Scale bars in **b**: 50 µm. **d**, **e** Immunostaining of chCOs and control hCOs on D45 for GFAP. Shown in **e** is the quantification of the percentage of the GFAP-positive area over the whole organoid area. Data represent the mean ± s.e.m. (*n* = 7 organoids, from three different batches; **p < 0.01). Scale bars in **d**: left image 100 µm, right image 30 µm for hCOs and chCOs, respectively. **f**, **g** Immunostaining of chCOs and control hCOs on D45 for S100B. Shown in **g** is the quantification of the percentage of the S100B-positive area over the whole organoid area. Data represent the mean ± s.e.m. (*n* = 8 organoids, from three different batches; **p < 0.01). **h** Flow cytometry analysis of CD44 expression of chCO and control hCO cells. **i** Immunostaining of astrocyte-lineage cells purified from chCOs on D45 for GFAP and S100B. Scale bars, 20 µm. **j** qPCR analysis of mature and fetal astrocyte gene expression of chCO and control hCO cells. Data represent the mean ± s.e.m. (*n* = 5 different batches; *p < 0.05, **p < 0.01)

We hypothesized that expression of the astrogenic factors induce astrocyte differentiation in human cerebral organoids. We first introduced the astrogenic factors NFIB and SOX9 into induced pluripotent stem cells (iPSCs) to accelerate the induction of astrocytes. The immunuostaining of NFIB and SOX9 showed that nearly all cells were transduced, although no selection procedure is applied (Supplementary Fig. 1a). Then the above induced iPSCs were mixed and cocultured with noninfected iPSCs under the standard condition of cerebral organoids to generate the chCOs (Fig. 1a). We next evaluated the astrocytic differentiation in these chCOs and control hCOs. By immunostaining, we found that GFAP^+^ astrocytes already appeared in chCOs at day 20 (D20) while very few GFAP^+^ cells were detected in control hCOs (Fig. 1b, c). By extending the time of culturing to D45, we observed that the ratio of GFAP^+^ astrocytes in chCOs further increased to 10~15%, and these cells got longer in morphology (Fig. 1d, e). In addition, we examined another astrocyte-specific marker S100B by immunostaining, and found that S100B^+^ cells also significantly increased in chCOs compared with control hCOs (Fig. 1f, g). Efficient induction of CD44 (cell surface marker for astrocytes) in chCOs was also confirmed by flow cytometry analysis (Fig. 1h). We also purified astrocyte-lineage cells from chCOs on day 45 by immunopanning and found that these isolated cells expressed GFAP as well as astrocyte marker S100B (Fig. 1i). In agreement with immunostaining and FACS results, qRT-PCR analysis revealed a robust increase in expression of astrocyte markers (*GFAP*, *S100B* and *GLAST*) and mature astrocyte genes (*AGXT2L1*, *RANBP3L*, *IGFBP7*, *GLT1* and *ALDOC*) in chCOs on day 45 (Supplementary Fig. 1b, j).^20^ However, no significant alteration in fetal astrocyte genes was observed, since the increased number of all astrocytes in chCOs may possibly result in comparable number of fetal astrocytes in chCOs and hCOs even though these fetal astrocytes comprise a smaller percentage of total astrocytes in chCOs (Fig. 1j). In addition, to verify the efficiency of astrocyte generation, we co-stained of SOX9 with GFAP and MAP2, and found that nearly 90% SOX9^+^ cells were GFAP^+^, while only ~8% SOX9^+^ cells were MAP2^+^, suggesting that most transduced iPSCs differentiated to astrocytes (Supplementary Fig. 1c). Collectively, our results indicated that astrogliogenesis was accelerated in chCOs.

### Enhanced neurogenesis and neuronal maturation in chCOs

**Fig. 2:**
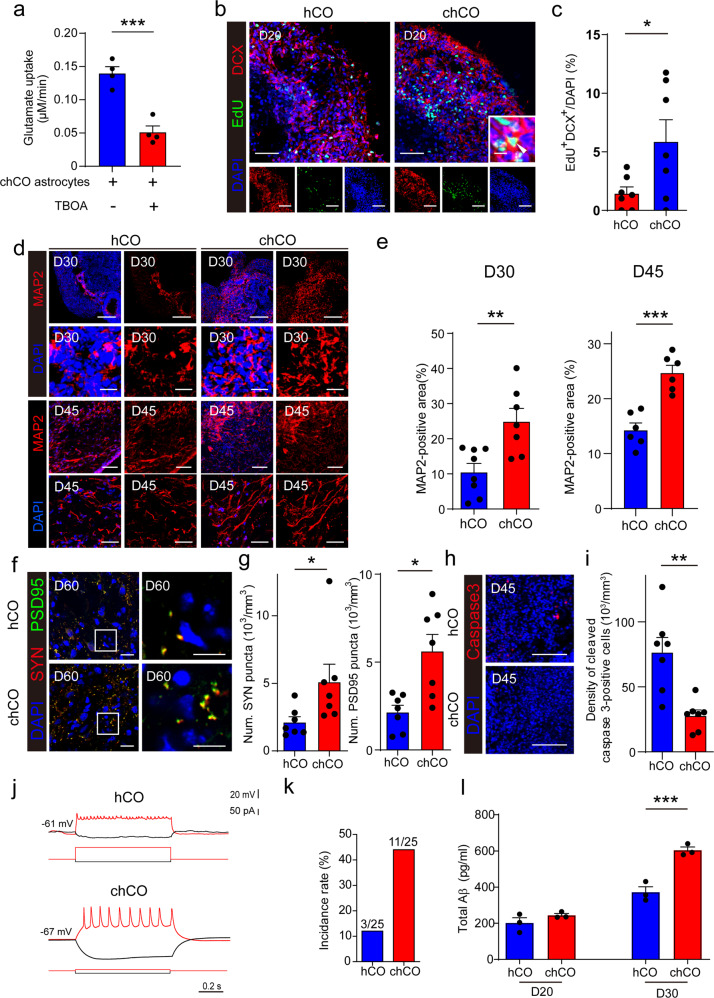
Functional analysis of induced astrocytes in human brain organoids. **a** Glutamate uptake by astrocyte purified from D45 chCOs. Data represent the mean ± s.e.m. (*n* = 4 different batches; ***p < 0.001). **b**, **c** Immunostaining of chCOs and control hCOs on D20 for EdU and DCX. Shown in **c** is the quantification of the density of the EdU/DCX-double-positive cells. Data represent the mean ± s.e.m. (*n* = 7 organoids, from three different batches; *p < 0.05). Scale bar in **a**: 50 µm. **d**, **e** Immunostaining of chCOs and control hCOs on D30 and D45 for MAP2. Shown in **e** is the quantification of the percentage of the MAP2-positive area over the whole organoid area. Data represent the mean ± s.e.m. (*n* = 6–8 organoids, from three different batches; **p < 0.01, **p < 0.001). Scale bars in **d**: upper panel 100 µm, lower panel 20 µm (D20); upper panel 100 µm, lower panel 50 µm (D45); **f**, **g** Immunostaining of chCOs and control hCOs on D60 for synaptophysin (SYN) and PSD95. Shown in **g** is the quantification of the density of the SYN or PSD95-positive puncta over the whole organoid area. Data represent the mean ± s.e.m. (*n* = 7 organoids, from three different batches; *p < 0.05). Scale bars in **e**: left panel 20 µm, right panel 10 µm. **h**, **i** Immunostaining of chCOs and control hCOs on D45 for cleaved caspase-3. Shown in **i** is the quantification of the density of the cleaved caspase-3-positive cells. Data represent the mean ± s.e.m. (*n* = 7 organoids, from three different batches; **p < 0.01). Scale bars in **g**: 50 µm. **j** Current clamp recordings of one cell in chCOs and control hCOs on D60. **k** Action potential (AP) of chCOs and control hCOs on D60. **l** Total Aβ levels in the supernatants of chCOs and control hCOs on D20 and D30. Data represent the mean ± s.e.m. (*n* = 3 different batches; ***p < 0.001)

Astrocytes maintain brain homeostasis by clearing neurotransmitters such as glutamate. To determine whether astrocytes from chCOs enable neurotransmitter clearing, we measured glutamate uptake by astrocytes purified from D45 chCOs. These astrocytes took up glutamate at a rate of about 0.14 µM/min, which was largely reduced by DL-threo-β-benzyloxyaspartic acid (TBOA, a competitive blocker of excitatory amino acid transporters) treatment (Fig. 2a), suggesting astrocytes from chCOs enabled neurotransmitter clearing. Astrocytes contributes to the process of neurogenesis.^23^ To analyze the levels of neurogenesis in chCOs and control hCOs, we labeled the dividing neural progenitor cells by 5-ethynyl-2’-deoxyuridine (EdU) and analyzed their neuronal differentiation 5 days after EdU labeling by co-stained the marker of newborn neurons doublecortin (DCX). We found that the density of newborn neurons, marked as EdU/DCX-double-positive cells, was increased significantly in chCOs, suggesting that the neurogenesis process was promoted in chCOs (Fig. 2b, c). Consistently, we also observed a marked increase in the number of MAP2^+^ neurons in chCO on D30 and D45 (Fig. 2d, e), suggesting elevated neurogenesis in these organoids. We next assessed whether the synapse formation was enhanced in chCOs by immunostaining for the pre- and postsynaptic proteins Synapsin I (SYN) and PSD95, the results showed that chCOs exhibited more synaptophysin and PSD95 puncta than control hCOs, indicating the increased number of astrocytes enhanced synapse formation in chCOs (Fig. 2f, g). Besides, less apoptotic cells were observed in chCOs by immunostaining with cleaved caspase-3 antibody, suggesting improved cell viability of chCOs (Fig. 2h, i). To further compare the difference between chCOs and hCOs in neuronal maturation, we performed electrophysiological recordings to characterize neuronal activity. Electrophysiological analysis with whole-cell recording in slices from D60 chCOs showed that 11 of 25 cells produced multiple high-amplitude action potentials (APs). In contrast, only 3 of 25 cells from D60 control hCOs produced low-amplitude APs, which is indicative of more mature neurons in chCOs (Fig. 2j, k). Since it was reported that accelerated neuronal maturation increased AD-associated Aβ secretion, we measured the level of secreted Aβ in chCO and hCO cultures by enzyme-linked immunosorbent assay (ELISA).^24^ In consistent with their results, we found that the Aβ level was higher in D30 organoid cultures compared with their D20 counterparts. Notably, there was a larger increase of Aβ level in chCO supernatant while extending the time of culturing from D20 to D30, supporting the accelerated neuronal maturation in chCOs (Fig. 2l). In conclusion, these results indicated enhanced neurogenesis and neuronal maturation in chCOs.

### *APOE4* chCOs exhibit elevated levels of lipid droplets, Aβ and p-tau than *APOE4* hCOs

We next determined whether increased proportion of astrocytes in chCOs affected AD modeling. First, *APOE4* induced pluripotent stem cells (iPSCs) were generated from their parental *APOE3* cells by CRISPR/Cas9 gene editing. Then the *APOE4* iPSCs and their *APOE3* parental cells were used to generate hCOs or chCOs with *APOE3* or *APOE4* genotype. We analyzed lipid droplet content in these organoids by nile red staining and found that *APOE4* increased lipid droplet levels in both hCOs and chCOs compared with their *APOE3* counterparts, and *APOE4* chCOs showed more lipid droplets than *APOE4* hCOs (Supplementary Fig. 2a, b). Similar alterations were also observed in Aβ and p-tau levels (Supplementary Fig. 2c, d). Taken together, these results indicated that chCOs exhibited elevated level of disease-associated pathologies than conventional hCOs while modeling AD.

### Astrocytic and neuronal *APOE4* elevate cholesterol and lipid droplets in neurons

**Fig. 3:**
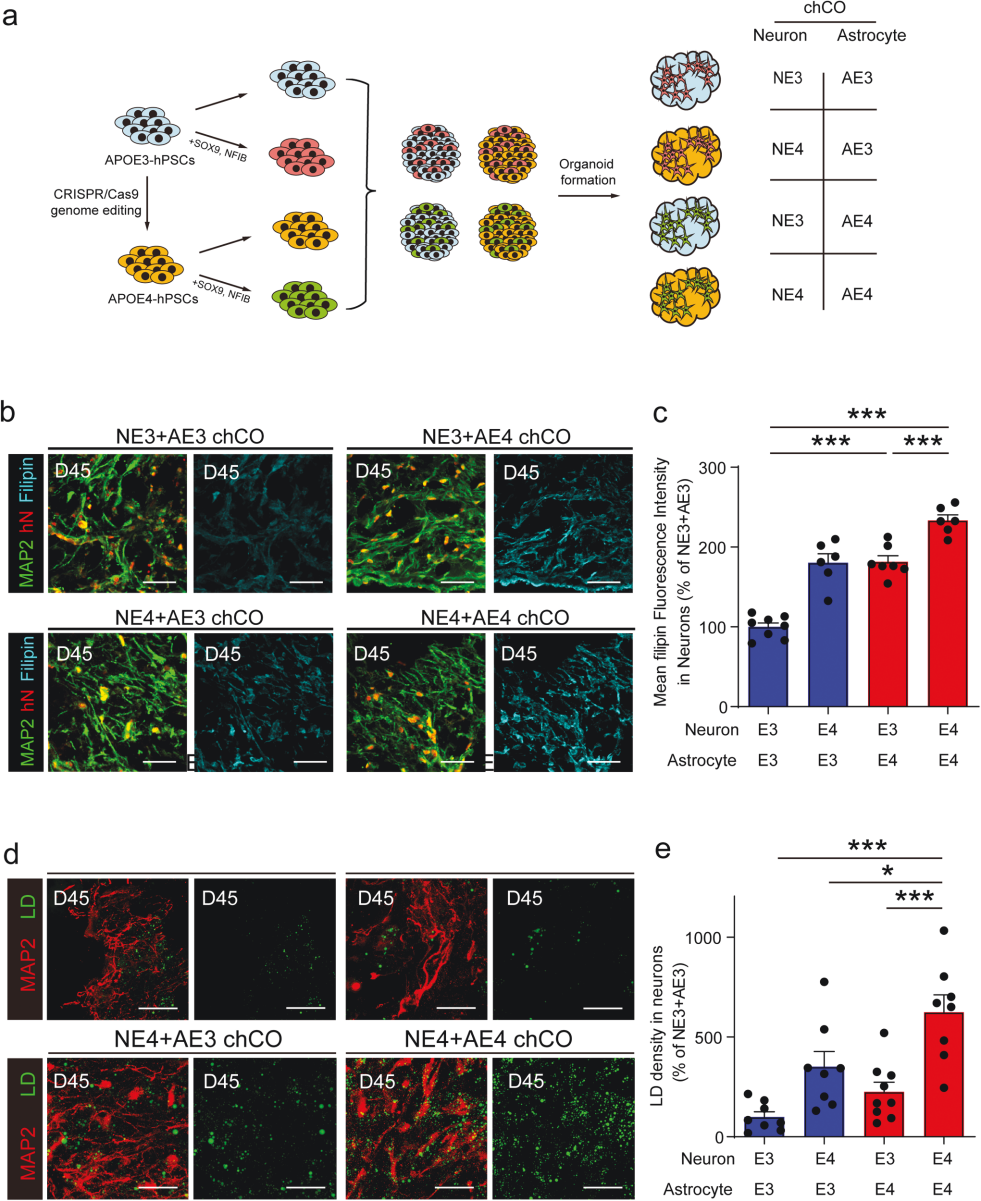
Astrocytic and neuronal *APOE4* elevated cholesterol and lipid droplet levels in neurons. **a** Experiment procedure for generation of chCOs with neurons and astrocytes carrying *APOE3* or *APOE4* allele. **b** Representative images of different chCOs on D45 stained with filipin, MAP2, and human nuclei. Scale bar: 50 µm. **c** Quantification of mean filipin fluorescence density in the neurons. Data represent the mean ± s.e.m. (*n* = 6–8 organoids, from three different batches; ***p < 0.001). **d** Representative images of chCOs and control hCOs on D45 stained with LipidTox and MAP2. Scale bar: 30 µm. **e** Quantification of the lipid droplet density in the neurons. Data represent the mean ± s.e.m. (*n* = 8–9 organoids, from four different batches; *p < 0.05, ***p < 0.001)

Our results showed that astrocytes barely appeared in D45 hCOs, so that most astrocytes in chCOs originated from NFIB/SOX9 virus-infected iPSCs. To address the distinct role of neuronal and astrocytic *APOE4*, we generated four kinds of chCOs with different *APOE* genotypes in neurons and astrocytes by mixing NFIB/SOX9 virus-infected and noninfected *APOE3* or *APOE4* iPSCs (Fig. 3a). The percentage of MAP2^+^ and GFAP^+^ cell was similar between differentiation kinds of chCOs, indicating that neuron/astrocyte differentiation in these organoids was not affected by *APOE* genotype (Supplementary Fig. 3a, b). We also verified *APOE3* and *APOE4* expression in chCOs or neurons purified from them by real-time PCR with published *APOE4* and *APOE3*-specific primers (Supplementary Fig. 3c, d).^25,26^ The identities of the neurons in chCOs were analyzed by by immunostaining with vGLUT1 (glutamatergic), GAD1 (GABAergic) and TH (dopaminergic). The results showed that there were more vGLUT1-positive cells than GAD1-positive or TH-positive cells in chCOs (Supplementary Fig. 3e, f). The neuronal cholesterol levels in these chCOs were analyzed by filipin staining. We noticed that astrocytic or neuronal *APOE4* alone was able to elevate the cholesterol levels in neurons, while both of them contributed to the maximum effect, indicating that astrocytic and neuronal ApoE is involved in the regulation of neuronal cholesterol homeostasis (Fig. 3b, c). We also detected neuronal lipid droplet content in the chCOs and similar results were observed (Fig. 3d, e). Overall, these results showed that both astrocytic and neuronal *APOE4* exacerbated the lipid burden in neurons.

### Astrocytic and neuronal *APOE4* are both indispensable for the elevation of neuronal p-tau levels in chCOs

**Fig. 4:**
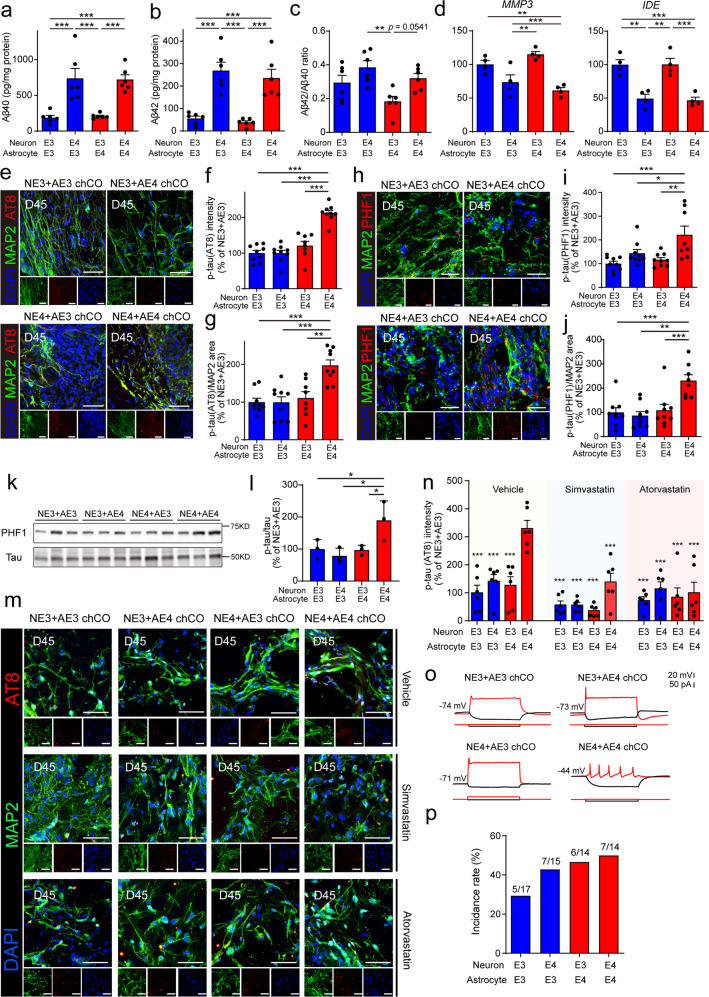
The co-occurrence of A*POE4* astrocytes and neurons are required for the elevation of neuronal p-tau levels. **a** Aβ40 levels in the supernatants of different chCOs on D45. Data represent the mean ± s.e.m. (*n* = 6 different batches; ***p < 0.001). **b** Aβ42 levels in the supernatants of different chCOs on D45. Data represent the mean ± s.e.m. (*n* = 6 different batches; ***p < 0.001). **c** The ratio of Aβ42/ Aβ40 in the supernatants of different chCOs on D45. Data represent the mean ± s.e.m. (*n* = 6 different batches; **p < 0.01). **d** qPCR analysis of Aβ depredating enzymes expression in different chCOs. Data represent the mean ± s.e.m. (*n* = 5 different batches; **p < 0.01, ***p < 0.001). **e** Immunostaining of different chCOs on D45 for p-tau (AT8) and MAP2. Scale bar: 50 µm. **f** Quantification of the p-tau (AT8) intensity in the neurons. Data represent the mean ± s.e.m. (*n* = 8–9 organoids, from three different batches; ***p < 0.001). **g** The percentage of the p-tau (AT8)-positive area in the neurons. Data represent the mean ± s.e.m. (*n* = 8–9 organoids, from three different batches; **p < 0.01, ***p < 0.001). **h** Immunostaining of different chCOs on D45 for p-tau (PHF1) and TUJ1. Scale bar: 30 µm. **i** Quantification of the p-tau (PHF1) intensity in the neurons. Data represent the mean ± s.e.m. (*n* = 8–9 organoids, from three different batches; *p < 0.05, **p < 0.01, ***p < 0.001). **j** The percentage of the p-tau (PHF1)-positive area in the neurons. Data represent the mean ± s.e.m. (*n* = 8–9 organoids, from three different batches; **p < 0.01, ***p < 0.001). **k** p-tau (PHF1) levels of different chCOs on D45 analyzed by western blotting. **l** Quantification of p-tau (PHF1) levels normalized to total tau. Data represent the mean ± s.e.m. (*n* = 3 different batches). **m** chCOs were analyzed for p-tau (AT8) and MAP2 after 10-day-treatment of simvastatin (10 µM) and atorvastatin (10 µM) (D35-D45). Scale bar: 30 µm. **n** Quantification of the p-tau (AT8) intensity in the neurons of chCOs from **m**. Data represent the mean ± s.e.m. (*n* = 6 organoids, from three different batches; ***p < 0.001, compared with NE4 + AE4 vehicle group). **o** Current clamp recordings of one cell in different chCOs on D60. **p** Action potential (AP) of different chCOs on D60

Next, we investigated the Aβ and tau pathology in these four kinds of chCOs. ELISA analyses of different Aβ species in the D45 chCO culture supernatants revealed that compared with chCOs containing *APOE3* neurons and *APOE3* astrocytes (NE3 + AE3), the levels of Aβ40 and Aβ42 were significantly increased in chCOs containing *APOE4* neurons (NE4 + AE3 and NE4 + AE4). However, no obvious elevation of Aβ levels was observed in chCOs containing *APOE3* neurons and *APOE4* astrocytes (NE3 + AE4), suggesting that *APOE4* neurons were required for alteration of Aβ levels (Fig. 4a, b, c). In addition, neither astrocytic nor neuronal *APOE4* affected the protein levels of immature and mature APP, indicating that they exerted little effect on expression regulation of APP (Supplementary Fig. 4a, b, c). Similarly, no significant changes in the levels of Nicastrin (NCT), ADAM Metallopeptidase Domain 10 (ADAM10) and beta-secretase1 (BACE1) in chCOs were observed, suggesting that secretases were not altered in these chCOs (Supplementary Fig. 4d, e, f, g). We also examine the expression of Aβ depredating enzymes in four kinds of chCOs and found that *MMP3* and *IDE* expression were reduced in NE4 + AE3 and NE4 + AE4 chCOs, suggesting that Aβ clearing mechanism may be impaired in these chCOs (Fig. 4d and Supplementary Fig. 4h). To examine the tau pathology in these chCOs, we co-stained them with anti-p-tau (AT8) and anti-MAP2 antibodies and found that there was no significant difference in neuronal p-tau levels between NE3 + AE3, NE3 + AE4 and NE4 + AE3 chCOs. However, the neuronal p-tau levels were significantly increased in NE4 + AE4 chCOs compared with the other three kinds of chCOs, suggesting that astrocytic and neuronal *APOE4* were both required for elevated neuronal tau phosphorylation (Fig. 4e, f, g). Similar results were obtained by immunostaining with another p-tau antibody (PHF1) (Fig. 4h, i, j). The change in p-tau levels was also verified by western blotting (Fig. 4k, l). It is reported that excess cholesterol is related to Alzheimer’s Aβ and tau pathologies.^27,28^ Thus, we hypothesize that cholesterol may mediates the effect of *APOE* on tau pathology in chCOs. To test this, we treated chCOs with two inhibitors of cholesterol synthesis: atorvastatin and simvastatin. The results demonstrate that atorvastatin and simvastatin both significantly reduce the levels of p-tau in NE4 + AE4 chCOs, indicating that *APOE4* may induce tau pathology by enhancing cholesterol level (Fig. 4m, n). Besides, we also performed functional studies in these chCOs including electrophysiological recordings and synapse formation analysis. The SYN and PSD95 staining were similar between different kinds of chCOs, and the frequency of action potentials between these four kinds of chCOs was also not significant (Fig. 4o, p and Supplementary Fig. 4i, j). To validate the findings from iPSC-derived chCOs, we also examined the Aβ and tau levels in H9-ESC-derived chCOs and similar results were obtained (Fig. S4k, l, m, n, o). Altogether, these results indicated that *APOE4* neurons were required for alteration of Aβ levels, while the elevation of neuronal p-tau required both astrocytic and neuronal *APOE4*.

## Discussion

Three-dimensional human cerebral organoid culture provides a novel system to study the pathogenesis of AD and to evaluate potential therapeutic interventions. In order to better recapitulate the features of neurological diseases including AD, efforts have been made to improve hCO generation. Astrocytes are critically involved in the pathogenesis of AD, however their differentiation and maturation in hCOs is slow compared with neurons.^20,21^ In the study, we developed a protocol to boost astrogliogenesis in part of chCOs by virus-mediated expression of two gliogenic transcription factors NFIB and SOX9. The virus-mediated expression of gliogenic transcription factors NFIB and SOX9 has been applied for rapid and reproducible acquisition of astrocytes in two-dimensional (2D) culture.^29^ However, they are not good representation of the complicated in vivo environment. chCOs allow more interactions between different neural cells and provide better spatial architectures, but they also have limitations, such as batch-to-batch variability and difficulty for downstream analysis. Astrocytes are known to play a critical role in the formation of neuronal networks.^23,30^ Our data demonstrates accelerated generation of functional astrocytes as well as more immature and mature neurons in chCOs, suggesting that the increased number of astrocytes may promote the neuronal differentiation and maturation in chCOs. The findings indicate that chCOs could serve as a useful platform to study neurological diseases involving both neurons and astrocytes. Cakir et al.^31^ reported that ectopic expression of ETV2 drove vascularization in hCOs. Our study together with their findings suggest that forced expression of lineage-specific differentiating factors is a promising approach to improve the organoid generation for complex organs such as brain, heart and liver. Microglia-astrocyte crosstalk and microglia-neuron crosstalk have been reported to be critical in the pathology of AD by modulating clearance of Aβ/tau and neuronal survival.^32–34^ Since microglia originate from the mesoderm lineage and other CNS cells are derived from neuroectodermal progenitors, most cerebral organoids generated by classical protocols lack the integration of microglia. Ormel et al.^35^ reported that microglia developed in cerebral organoids by in the absence of SMAD inhibition. Following their findings, we will further optimize the current protocol to develop microglia in chCOs. Moreover, with these chimeric organoids, we are able to assess the intricate and cell type-dependent effects of various disease risk genes in a more physiologically relevant context.

*APOE4* has a profound impact on dysregulation of lipid metabolism associated with AD pathogenesis. We observed that the levels of neuronal lipid droplets and cholesterol were higher in chCOs with *APOE4* astrocytes than those with *APOE3* astrocytes, suggesting that *APOE4* astrocytes were associated with dysregulated lipid and cholesterol in neurons. Consistent with our findings in chCOs, a recent study has also reported diminished capacity of *APOE4* astrocytes in eliminating neuronal lipids.^14^

Studies have reported that *APOE4* worsens tau pathology in both animal and human cell models.^13,36^ Selective removal of astrocytic *APOE4* in the P301S tau/apoE4 mice reduces cortical and hippocampal phosphorylated tau levels, suggesting an important role of astrocytic *APOE4* in tau pathology.^15^ Moreover, co-culturing P301S tau expressing-neurons with *APOE4* astrocytes also significantly elevates neuronal tau phosphorylation and cell death.^37^ Consistently, although the neurons didn’t express pathological tau mutants, we were able to observe increased neuronal p-tau levels in chCOs containing both *APOE4* neurons and astrocytes. However, the raise in neuronal levels of p-tau was not detected in other chCOs. These results suggested that neuronal and astrocytic *APOE4* were both critical for the increased phosphorylation of tau. Notably, a recent study demonstrates that neuronal tau can be transported to astrocytes in vivo.^38^ In addition, ApoE is observed to interact with tau in an allele-dependent manner.^39^ These findings together with our data support the idea that the phosphorylation process of tau in neurons may be accelerated by *APOE4* astrocytes, and the neuron-astrocyte transportation of p-tau is possibly impaired by neuronal *APOE4*. Besides, *APOE2* is reported to exert protective effects against AD, such as reducing APP transcription and Aβ secretion.^40,41^ However, it is also associated with increased tau pathology, suggesting the intricate role of ApoE2 in regulating tau and Aβ.^42^ It will be very interesting to verify the effect of *APOE2* neurons or astrocytes on AD-associated pathologies in chCOs. Morever, in consistant with other studies in 2D culture, we observed that the ptau level was also significantly reduced in in NE4 + AE4 chCOs after inhibiting cholesterol synthesis by atorvastatin and simvastatin, suggesting targeting cholesterol metabolism is a valuable strategy in developing therapeutic agents for AD patients carrying *APOE4.*^28^ Wang et al.^27^ recently report that cholesterol is synthesized in astrocytes and transferred to neurons in apoE lipoprotein particles. These cholesterol intergates into the neuronal membranes and forms lipid rafts to enhancing the interation of APP with β- and γ-secretase, thus promotes Aβ production. In additon to their findings, our results showed that neither astrocytic nor neuronal *APOE4* affected the expression of APP and secreastes. Meanwhile, expression of some Aβ depredating enzymes in neurons were reduced in NE4 + AE3 and NE4 + AE4 chCOs, suggesting that Aβ clearing mechanism may be impaired by *APOE4*. Taken together, these findings indicates that apoE may paticipate in multiple steps of Aβ metabolism, and further investigation on the underlying molecular mechanism is intriguing. Given the essential role of *APOE4* in the pathogenesis of AD, our findings not only provide a deeper understanding between different *APOE4* cells and tauopathy, but also help to develop potential therapeutic strategies for patients who carry the *APOE4* allele.

## Materials and methods

### hPSC culture

The human induced PSC line were generated from a 66-year-old healthy female by iXCells Biotechnologies (catalog no. 30HU-002) and cultured on Matrigel (BD Biosciences) coated dishes in mTeSR1 medium (Stem Cell Technologies). Cultures were passaged every 5–7 days with Gentle Cell Dissociation Reagent (Stem Cell Technologies). The APOE3 and APOE4 H9-ESCs were kindly provided by Dr. Ru Zhang, Tongji University. All cells were confirmed negative for mycoplasma.

### Generation of chimeric human cerebral organoids (chCOs) with *NFIB* and *SOX9* induction

STEMdiff™ Cerebral Organoid Kit (Stem Cell Technologies) was applied for generation of chCOs. The hPSCs or H9-ESCs were dissociated with Gentle Cell Dissociation Reagent (Stem Cell Technologies), infected with FUGW-NFIB and FUGW-SOX9 lentivirus, mixed with equal amount of uninfected hPSCs (4500 infected cells and 4500 uninfected cells), and plated into each well of a 96-well round-bottom ultra-low-attachment plate containing EB Formation Medium to generate EBs. On day 5, EBs were transferred into a 24-well ultra-low attachment plate containing Induction Medium and cultured for an additional 2 days. After embedded in Matrigel, these organoids transferred to Expansion Medium, and cultured for 3 days. On day 10, they were transferred to Maturation Medium and placed on an orbital shaker for extended periods of culture.

### Immunofluorescence staining and quantification

Remaining Matrigel was removed by washing with phosphate-buffered saline (PBS) before fixation. Then organoids were fixed with 4% PFA in PBS for 30 min, washed three time with PBS, and submerged in 30% sucrose for 1–2 days until samples sank. After embedded in OCT, organoids were cryosectioned at 20 μm. Sections were incubated overnight at 4 °C with primary antibodies diluted in PBS containing 0.3% Triton X-100 and 3% donkey serum. After washing with PBS for three times, sections were incubated with secondary antibodies at room temperature for 1 h. The information of primary antibodies used were as follows: GFAP (rabbit, DAKO Z033401, 1:1000),MAP2 (rabbit, Millipore AB5622, 1:500), S100B (rabbit,BeyotimeAF1945, 1:200), DCX (goat, Santa cruz sc-8066, 1:200), SYN1 (rabbit, Synaptic systems 106103, 1:500), PSD95 (mouse, Abcam ab2723, 1:500), Cleaved Caspase-3 (rabbit, Cell signaling 9664, 1:500), AT8 (mouse, Thermo Fisher Scientific MN1020, 1:500), PHF1(rabbit, Abcam ab184951, 1:500), hNuclei (mouse, Milipore MAB1281, 1:200), NFIB (rabbit, Abcam ab186738, 1:100), SOX9 (mouse, Abcam ab76997, 1:100). Secondary antibodies used were as follows: donkey anti-rabbit cy3 (Jackson ImmunoResearch 711-165-152, 1:1000), donkey anti-rabbit Alexa 488 (Molecular probesA21206, 1:1000), donkey anti-mouse cy3 (Molecular Probes 715-165-150, 1:1000), donkey anti-goat cy3 (Jackson ImmunoResearch 705-165-147, 1:1000). For filipin staining, sections were washed three times with PBS and incubated with 1.5 mg/ml glycine for 10 min at RT. Then sections were stained with 0.05 mg/ml filipin (MCE, HY-N6716) in PBS containing 10% FBS for 2 h and rinsed three times with PBS. For lipid droplet staining, sections were washed three times with PBS and stained with LipidTox (Thermo Fisher Scientific, H34477) according to the manufacturer’s protocols. EdU staining were performed with BeyoClick EdU Cell Proliferation Kit with Alexa Fluor 488(Beyotime, C0071S) according to the manufacturer’s protocols. All images were acquired with Olympus FV10i confocal microscope. ImageJ software was used to quantify the images. For neuronal filipin, LipidTox, AT8 and PHF1 measurements, MAP2^+^ or TUJ1^+^ area was set as ROI, and the signals within ROI were analyzed.

### Enzyme-linked immunosorbent assay (ELISA)

The levels of total Aβ, Aβ40, and Aβ42 in supernatants of organoid cultures were measured by Human β Amyloid Total ELISA Kit (ExCell Bio, EH025-48), Human β Amyloid 40 ELISA Kit (ExCell Bio, EH039-96), and Human β Amyloid 42 ELISA Kit (ExCell Bio, EH040-96) according to the manufacturer’s instructions.

### Electrophysiological recordings

The D60 organoids were sliced into 200 µm thick sections with a vibratome in chilled oxygenated medium containing (in mM) 85 NaCl, 75 surcose, 2.5 KCl, 25 glucose, 1.25 NaH_2_PO_4_, 4 MgCl_2_, 0.5 CaCl_2_ and 24 NaHCO_3_.These sections were kept at RT before whole-cell patch-clamp recordings. Recordings were performed in aCSF containing (in mM) 126 NaCl, 2.5 KCl, 26 NaHCO_3_, 2CaCl_2_, 2 MgCl_2_, 1.25 NaH_2_PO_4_and 10 glucose saturated with 95% O_2_/5% CO_2_. Slices were viewed using an Olympus BX61WI upright microscope with infrared differential interference contrast optics and 60x water immersion objective. Glass recording pipettes (4–8 MΩ resistance) were filled with an intracellular solution composed of the following (in mM): 126 K-gluconate, 4 KCl,10 HEPES, 4 ATP-Mg, 0.3 Na_3_GTP and 10 Na-phosphocreatine. Recordings were acquired and analyzed using two Axon Multiclamp 700B amplifiers, Digidata 1440A (Molecular Devices, USA), and pCLAMP10 software (Molecular Devices, USA). Signals were sampled at 10 kHz with a 3 kHz low-pass filter.

### Flow cytometry

Organoids were dissociated into single cells with accutase. Then these cells were incubated with anti-CD44 PE antibody (Thermo Fisher Scientific, 12-0441-81, 0.125 µg per 1 × 10^5^ cells) for 30 min at 4 °C. Cells were washed and analyzed by Thermo Attune NxT.

### Purification of astrocytes and neurons from chCOs

Astrocytes and neurons were purified from chCOs by immunopanning following a published protocol.^14^ Briefly, 3–5 chCOs were chopped and digested with 30 U/ml papain. Then the cell suspension petri dishes, which were pre-coated with anti-Thy1 (for neurons, R&D, AF2067) or anti-HepaCAM (for astrocytes, R&D, MAB4108). The isolated neurons or astrocytes were detached by accutase incubation, then these cells were plated on poly-d-lysine coated coverslips for immunostaining, cultured in astrocyte medium (ScienCell, 1801) for glutamate uptake assay or lysed with TRIzol reagent (Sigma-Aldrich, T9424) for RNA isolation.

### Glutamate uptake

Purified astrocytes were seeded in a 96-well plate at a density of 5000 cells/well and cultured in astrocyte medium (ScienCell, 1801) for 5 days. The cells were incubated 30 min in Hank’s balanced salt solution (HBSS) buffer without calcium and magnesium, then incubated for 1 h in HBSS with calcium and magnesium containing 10 µM glutamate with or without DL-threo-β-Hydroxyaspartic acid (TBOA; 100 µM). The remaining glutamate within the supernatant was measured by Amplex™ Red Glutamic Acid/Glutamate Oxidase Assay Kit (Thermo Fisher Scientific, A12221) according to the manufacturer’s instructions.

### Real-time PCR

Total RNA was isolated from 2 to 3 organoids using TRIzol reagent (Sigma-Aldrich, T9424). cDNA was generated from 1 µg of RNA with PrimeScript RT master mix (TaKaRa, RR036A). Real-time PCR were performed with HotStart SYBR Green qPCR Master Mix (ExCell Bio, MB000-3012) on Mx3000P qPCR system (Agilent). Primer sequences were listed in Table S1.

### Western blotting

For western blotting, samples containing equivalent amount of protein were loaded into a 4–10% sodium dodecyl-sulfate polyacrylamide gel electrophoresis, and transferred to nitrocellulose membranes. After blocked with 4% BSA in TBST, the membranes were incubated primary antibodies overnight at 4 °C. Then the membranes were washed three times with TBST, and incubated with HRP-conjugated secondary antibodies 1 h at RT. Signal were detected by Clarity ECL western blotting substrates (BIO-RAD, 1705060) and analyzed by MiniChemi system (SAGECREATION). The information of primary antibodies used were as follows: APP (rabbit, Sigma-Aldrich 1:1000, A8717), PHF1(rabbit, Abcam ab184951, 1:500), tau (rabbit, ABclonal 1:1000, A0002), NCT (rabbit, Sigma-Aldrich 1:1000, N1660), ADAM10 (rabbit, Abcam 1:1000, ab1997), BACE1 (rabbit, Abgent 1:1000, AP7774b), β-actin (rabbit, Sigma-Aldrich 1:1000, A2066). Secondary antibody used was Goat anti-Rabbit IgG-HRP (Abmart 1:5000, M21002).

### Statistical analysis

Statistical analyses were performed with Prism 6 (GraphPad). Two-tailed unpaired Student’s *t*-test was used to compare the differences between hCOs and chCOs. Chi-square test was used to compare the differences of action potential between different organoids. For multiple comparisons, one-way analysis of variance (ANOVA) followed by Bonferroni test was used. A *p*-value less than 0.05 was considered statistically significant.

## Supplementary information

Supplementary Materials

## Data Availability

The datasets in this study are available from the corresponding author upon reasonable request.
