# Supplementary material for: Chimeric cerebral organoids reveal the essentials of neuronal and astrocytic APOE4 for Alzheimer’s tau pathology
Source: Signal Transduct Target Ther. 2022 Jun 13;7:176. doi: 10.1038/s41392-022-01006-x (PMC9189105; doi:10.1038/s41392-022-01006-x)
Supplement: Supplementary file 1 — Supplementary Materials [file 41392_2022_1006_MOESM1_ESM.docx]

Supplementary Materials for

Chimeric human cerebral organoids reveal the essentials of both neuronal and astrocytic *APOE4* for the Alzheimer’s tau pathology

Shichao Huang^1*^, Zhen Zhang^1^, Junwei Cao^2^, Yongchun Yu^2^ and Gang Pei^1,3,4*^

* Correspondence: [huangshichao@sibcb.ac.cn](mailto:huangshichao@sibcb.ac.cn) (S.H.), [gpei@sibs.ac.cn](mailto:gpei@sibs.ac.cn) (G.P.)

This file includes:

Figure. S1-S4

Table. S1

**
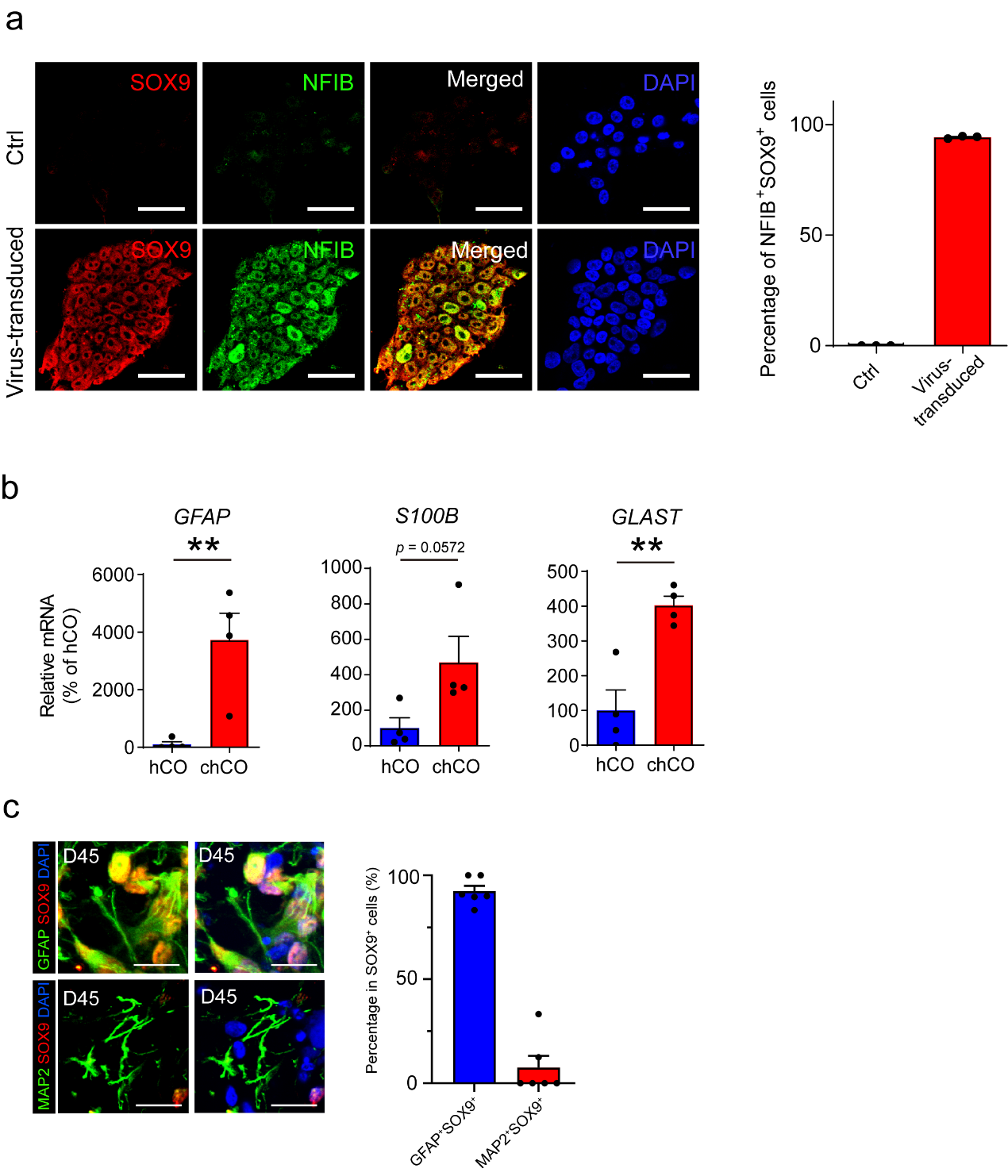
**

**Fig. S1. Characterization of hCOs and chCOs.**

**a.** Immunostaining of SOX9 and NFIB in control iPSCs or iPSCs transduced with virus. Scale bars, 50 µm. **(**n=4 different batches**)**

**b.** qPCR analysis of astrocyte marker expression of chCO and control hCO cells. Data represent the mean ±s.e.m. (n=4 different batches; ** p<0.01)

**c.** Immunostaining of SOX9 with GFAP or MAP2 in D45 chCOs. Scale bars, 20 µm. **(**n=6 organoids from 3 different batches**)**

**
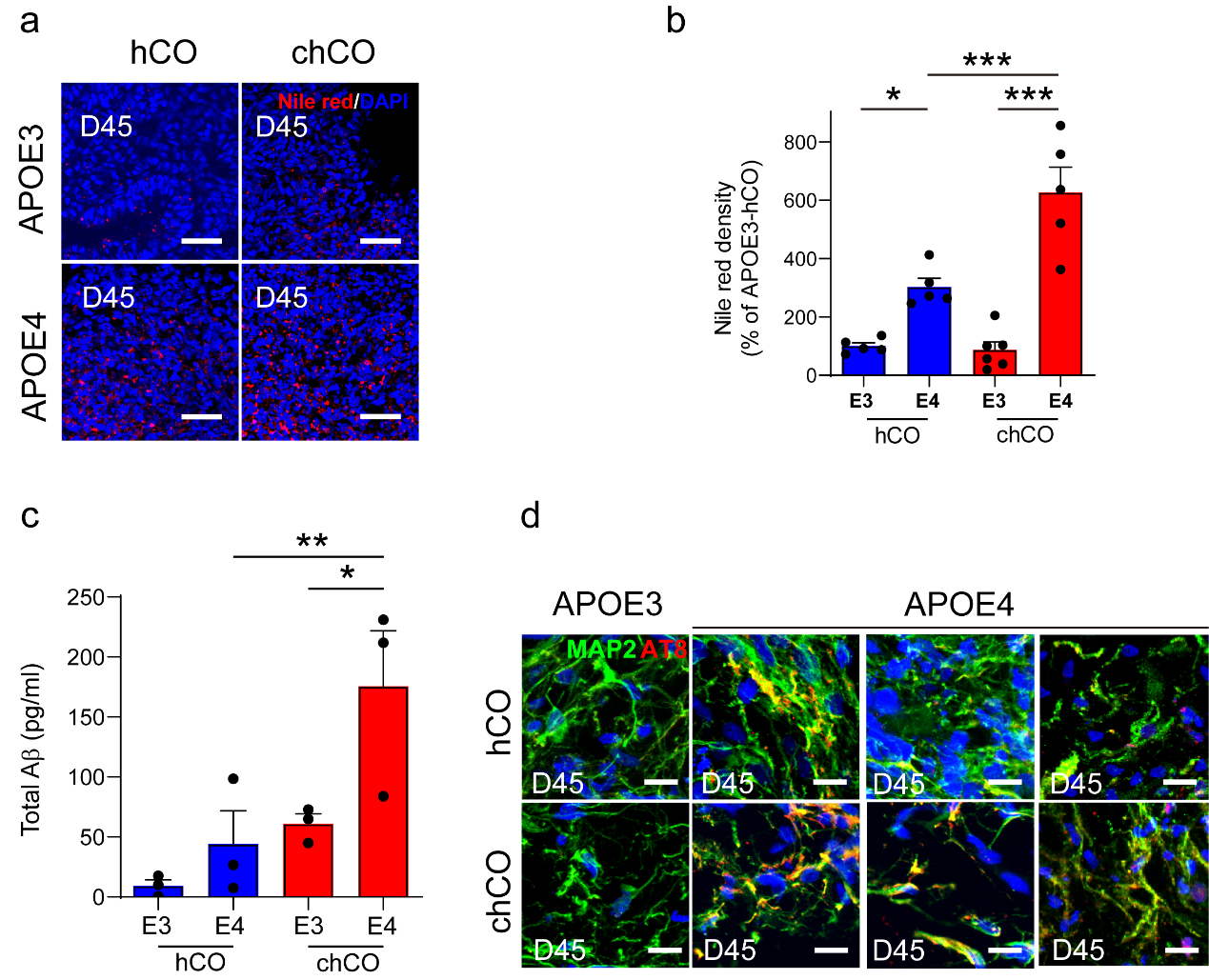
**

**Fig. S2. Evaluation of AD-associated pathologies in *APOE3* and *APOE4* chCOs.**

**a.** Representative images of chCOs and control hCOs on D45 stained with nile red. Scale bar: 50 µm.

**b.** Quantification of nile red density. Data represent the mean ± s.e.m. (n=5-6 organoids, from three different batches; * p<0.05, *** p<0.001)

**c.** Total Aβ levels in the supernatants of chCOs and control hCOs with *APOE3* or *APOE4* genotype. Data represent the mean ±s.e.m. (n=3 different batches; * p<0.05, ** p<0.01)

**d.** Immunostaining of chCOs and control hCOs with *APOE3* or *APOE4* genotype on D45 for p-tau (AT8) and MAP2. Scale bar: 20 µm.


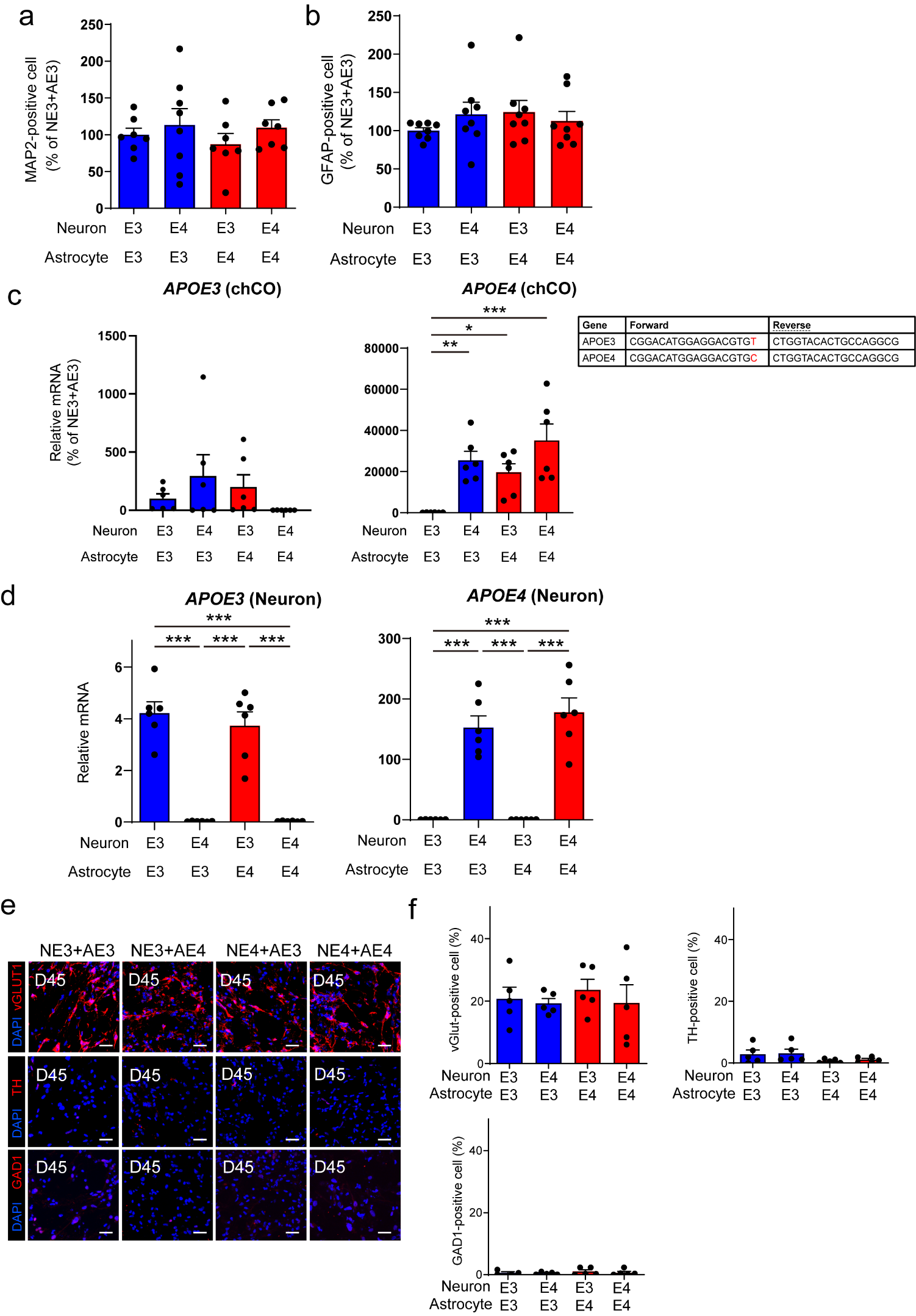


**Fig. S3. Characterization of different *APOE* chCOs.**

**a.** MAP2-positive cells in D45 chCOs. Data represent the mean ± s.e.m. (n=7-8 organoids, from three different batches)

**b.** GFAP-positive cells in D45 chCOs. Data represent the mean ± s.e.m. (n=8 organoids, from three different batches)

**c.** Real time PCR analysis of APOE3 and APOE4 expression in D45 chCOs. Data represent the mean ±s.e.m. (n=6 different batches, * p<0.05, ** p<0.01, *** p<0.001)

**d.** Real time PCR analysis of APOE3 and APOE4 expression in neurons purified from D45 chCOs. Data represent the mean ±s.e.m. (n=6 different batches, *** p<0.001)

**e.** Immunostaining of vGLUT1, TH or GAD1 in D45 chCOs.

**f.** Quantification of the percentage of the vGLUT1-positive, TH-positive or GAD1-positive area over the whole organoid area. Data represent the mean ± s.e.m. (n=4-5 organoids, from three different batches)

**
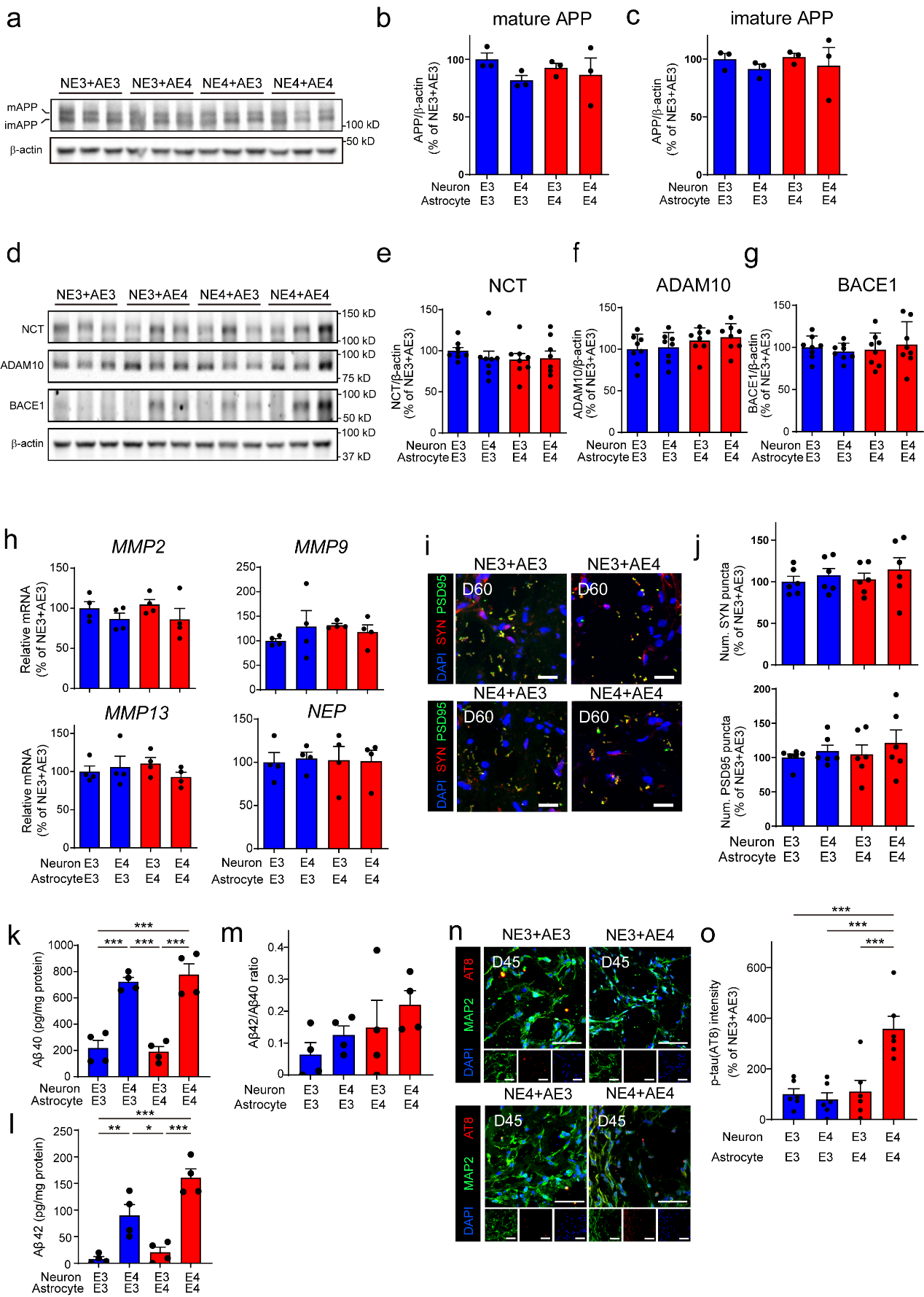
**

**Fig. S4. AD pathologies of different *APOE* chCOs.**

**a-c.** Mature and immature APP levels of different chCOs on D45 analyzed by western blotting. Shown in (b) and (c) are the quantification of mature and immature APP levels normalized to β-actin, respectively. Data represent the mean ±s.e.m. (n=3 different batches)

**d-g.** NCT, ADAM10 and BACE1 levels of different chCOs on D45 analyzed by western blotting. Shown in (e), (f) and (g) are the quantification of NCT, ADAM10 and BACE1 levels normalized to β-actin, respectively. Data represent the mean ±s.e.m. (n=3 different batches)

**h.** qPCR analysis of Aβ depredating enzymes expression in different chCOs. Data represent the mean ± s.e.m. (n=5 different batches)

**i-j.** Immunostaining of different chCOs on D60 for synaptophysin (SYN) and PSD95. Data represent the mean ± s.e.m. Scale bar: 10 µm. (n=6 organoids, from three different batches)

**k.** Aβ40 levels in the supernatants of different H9 ESC-derived chCOs on D45. Data represent the mean ±s.e.m. (n=4 different batches; *** p<0.001)

**l.** Aβ42 levels in the supernatants of different H9 ESC-derived chCOs on D45. Data represent the mean ±s.e.m. (n=4 different batches; * p<0.05, ** p<0.01, *** p<0.001)

**m.** The ratio of Aβ42/ Aβ40 in the supernatants of different H9 ESC-derived chCOs on D45. Data represent the mean ±s.e.m. (n=4 different batches)

**n.** Immunostaining of different H9 ESC-derived chCOs on D45 for p-tau (AT8) and MAP2. Scale bar: 50 µm.

**o.** Quantification of the p-tau (AT8) intensity in the neurons from different H9 ESC-derived chCOs. Data represent the mean ± s.e.m. (n=6 organoids, from three different batches; * p<0.05)

**Table S1. List of primer sequences used for real-time PCR analysis**

| **Gene** | **Forward** | **Reverse** |
| --- | --- | --- |
| APOE3 | CGGACATGGAGGACGTGT | CTGGTACACTGCCAGGCG |
| APOE4 | CGGACATGGAGGACGTGC | CTGGTACACTGCCAGGCG |
| AGXT2L1 | CAATGGGAAAACCGATGGGC | CCAACAGCACAAGATACTGGAT |
| RANBP3L | GACCTGCTATTTTGCAGCTACC | GCACCTAAAAAGTCCTGGTTAGT |
| IGFBP7 | CGAGCAAGGTCCTTCCATAGT | GGTGTCGGGATTCCGATGAC |
| GLT1 | CCTGACGGTGTTTGGTGTCAT | CAAGCGGCCACTAGCCTTAG |
| ALDOC | GCCAAATTGGGGTGGAAAACA | TTCACACGGTCATCAGCACTG |
| TOP2A | TTAATGCTGCGGACAACAAACA | CGACCACCTGTCACTTTCTTTT |
| TMSB15A | GGTCTCAGCCCCGCGAACAG | CAGGTAATGTCGAAATCTGCTGTTG |
| NNAT | ACTGGGTAGGATTCGCTTTTCG | ACACCTCACTTCTCGCAATGG |
| HIST1H3B | GTTGCTGATTCGGAAGCTGC | GAAGCGAAGATCGGTCTTGAA |
| MMP2 | TACAGGATCATTGGCTACACACC | GGTCACATCGCTCCAGACT |
| MMP3 | CTGGACTCCGACACTCTGGA | CAGGAAAGGTTCTGAAGTGACC |
| MMP9 | TGTACCGCTATGGTTACACTCG | GGCAGGGACAGTTGCTTCT |
| MMP14 | GGCTACAGCAATATGGCTACC | GATGGCCGCTGAGAGTGAC |
| NEP | AGAAATGCTTTCCGCAAGGCC | AGCCTC CCCACAGCATTTTCC |
| IDE | AGCAGGCTTGAGCTATGATCT | GTTCAGCCCGGAAATTGTTAAGA |
| GFAP | CTTTGCCAGCTACATCGAGA | ATTGTCCCTCTCAACCTCCA |
| S100B | CCAGCCGTGTTGTAGCTAAT | CAGCTTACACACAGGCCTAATA |
| GLAST | AGCAGGGAGTCCGTAAACG | AGCATTCCGAAACAGGTAACTTT |
